# Drug-Related Problems in Elderly Patients Attended to by Emergency Services

**DOI:** 10.3390/jcm13010003

**Published:** 2023-12-19

**Authors:** Jesús Ruiz-Ramos, Adrián Plaza-Diaz, Cristina Roure-i-Nuez, Jordi Fernández-Morató, Javier González-Bueno, María Teresa Barrera-Puigdollers, Milagros García-Peláez, Nuria Rudi-Sola, Marta Blázquez-Andión, Carla San-Martin-Paniello, Caterina Sampol-Mayol, Ana Juanes-Borrego

**Affiliations:** 1 Pharmacy Department, Hospital Santa Creu i Sant Pau, 08025 Barcelona, Spain; aplaza@santpau.cat (A.P.-D.); ajuanes@santpau.cat (A.J.-B.); 2 Department of Medicine, Universitat Autònoma de Barcelona, 08193 Barcelona, Spain; 3 Institut de Recerca Sant Pau (IR SANT PAU), 08041 Barcelona, Spain; mblazquez@santpau.cat; 4 Pharmacy Department, Consorci Sanitari de Terrassa, 08227 Terrassa, Spain; croure@cst.cat (C.R.-i.-N.); jfernandezm@cst.cat (J.F.-M.); 5 Pharmacy Department, Hospital Dos de Maig Consorci Sanitari Integral, 08025 Barcelona, Spain; javier.gonzalezbueno@sanitatintegral.org (J.G.-B.); mariateresa.barrera@sanitatintegral.org (M.T.B.-P.); 6 Central Catalonia Chronicity Research Group (C3RG), Universitat de Vic-Universitat Central de Catalunya, 08500 Vic, Spain; 7 Pharmacy Department, Hospital General de Granollers, 08402 Granollers, Spain; icosgp@gmail.com (M.G.-P.); nrudi@fhag.es (N.R.-S.); 8 Emergency Department, Hospital Santa Creu i Sant Pau, 08025 Barcelona, Spain; 9 Strategy and Innovation Office (Més Sant Pau), Hospital Santa Creu i Sant Pau, 08025 Barcelona, Spain; csanmartinp@santpau.cat (C.S.-M.-P.); csampol@santpau.cat (C.S.-M.)

**Keywords:** elderly, polypharmacy, emergency care, adverse events

## Abstract

The progressive aging and comorbidities of the population have led to an increase in the number of patients with polypharmacy attended to in the emergency department. Drug-related problems (DRPs) have become a major cause of admission to these units, as well as a high rate of short-term readmissions. Anticoagulants, antibiotics, antidiabetics, and opioids have been shown to be the most common drugs involved in this issue. Inappropriate polypharmacy has been pointed out as one of the major causes of these emergency visits. Different ways of conducting chronic medication reviews at discharge, primary care coordination, and phone contact with patients at discharge have been shown to reduce new hospitalizations and new emergency room visits due to DRPs, and they are key elements for improving the quality of care provided by emergency services.

## 1. Introduction

The progressive aging of the population and the increase in chronic conditions have led to a progressive increase in the number of drugs prescribed to the elderly population. Several studies have shown that the proportions of elderly patients with polypharmacy (>5 drugs) and severe polypharmacy (>10 drugs) have progressively increased during recent decades [1]. Due to reduced functional reserves and diminished renal and hepatic clearance, elderly patients are at a heightened risk of experiencing drug-related adverse events, with polypharmacy contributing to their frailty. Consequently, emergency departments (EDs) are encountering an increasing number of patients with polypharmacy [2], posing additional complexities in both diagnosis and preventing the exacerbation of chronic diseases during emergency room care. Conversely, visits to the ED are often prompted by severe and life-threatening conditions that can be complicated by hemodynamic instability, dehydration, or acute kidney and/or hepatic injury. Polypharmacy can exacerbate these factors, impacting individuals’ health outcomes.

Drug-related problems (DRPs), defined as injuries resulting from a medication taken for medical intervention, represent a significant public health concern for healthcare systems globally. It is estimated that approximately 5–10% of hospital admissions and between 10 and 30% of emergency department visits are attributable to DRPs, most of which are considered preventable. In recent decades, numerous studies have aimed to analyze the prevalence of DRPs as a cause for ED visits, revealing rates ranging between 10 and 30% [3,4]. DRPs often result in additional hospital days, higher rates of outpatient encounters, and increased cost of care [5]. Furthermore, it is well known that DRPs are commonly missed or underappreciated during an ED visit [6]. Anticoagulants, insulins, antiplatelet agents, and central nervous depressors are commonly used medications that have been implicated in DRPs requiring hospitalization [7].

Several studies have demonstrated the impact of in-hospital medication reviews on readmission rates, ED visits, length of hospital stay, and mortality [8,9]. While the effects of in-hospital medication reviews have been well-researched, the impact of medication reviews for older patients living with frailty in the ED has yet to be fully studied. This is despite the high number of drug-related ED visits and the inherent risks associated with the transition from emergency care to hospital treatment. ED care offers an opportunity to reconcile medications, screen for potentially inappropriate medication and drug interactions, mitigate the risks associated with inappropriate medication use, and even deprescribe unnecessary drugs when warranted.

The aim of this review is to describe the impact of polypharmacy in elderly patients admitted to the ED and to describe the experiences with the greatest amount of evidence available in order to prevent DRPs in these high-complexity patients.

## 2. Polypharmacy in Elderly Patients Attended to in the ED

Polypharmacy is a global public health issue [5]. However, there is still no universally accepted definition for the term. Most of the literature uses a numerical definition, setting the threshold for polypharmacy at five or more medications. However, it remains unclear whether over-the-counter medications are included or what the minimum treatment duration should be for counting [1].

In 2022, a systematic review encompassing 106 studies published between 1989 and 2019 revealed a polypharmacy prevalence of 37% [7]. This rate varies depending on the definition used, patients’ ages, and publication year. In our local context, the 2017 National Health Survey included 7023 participants with an average age of 76 years and reported a polypharmacy prevalence of 27.3%. Factors associated with this included the number of chronic diseases and the level of dependency on these drugs for basic daily activities [10]. A systematic review indicated a polypharmacy prevalence in elderly individuals ranging from 27% to 59% in primary care and 46% to 84% in hospitalized patients [11]. A European study found a prevalence of polypharmacy between 26.3% and 39.9%. Likewise, a 32% prevalence was observed in the population over 85 years old, which is consistent with data on primary care patients [12]. Regarding the ED, recent studies have shown an increase in patients with polypharmacy being attended to in these units. A multicenter study of elderly patients in Spain showed that 64,1% of the patients presented polypharmacy at ED admission, whereas 22% presented with more than 10 chronic drugs prescribed [13]. On the other hand, the complexity of chronic prescription treatment, which includes not only the number of drugs but also the number of medication intakes per day, the pharmaceutical forms, and the need to divide doses, plays a relevant role in ensuring an adequate understanding of the medication by the patients [14]. It has been observed that patients who consult the ED for RPDs and are discharged with more complex treatments have a higher risk of new consultations in the short term [15].

While polypharmacy figures vary depending on study criteria, it is evident that this problem is growing. This growth can be attributed to the aging population and the subsequent rise in multimorbidity with age. In Europe, the average life expectancy at 65 years is 20 years longer, with a population pyramid skewed toward the elderly, where 10% are aged 65 or older. By 2050, it is projected that up to 16% of Europe’s population will be over 65 years old [16]. Additionally, clinical guidelines for chronic diseases increasingly lower the threshold for starting pharmacological treatment, recommending a greater number of drugs for each condition [17]. However, there is a lack of practice guidelines for older patients with multiple pathologies that focus on the individual rather than isolated diseases. Consequently, most patients receive prescriptions from different specialists within a fragmented care model, which poses safety risks, especially for older, frail patients and other vulnerable groups such as those with severe mental health disorders or those at risk of social exclusion.

What is more intriguing than this numerical concept is a definition that, irrespective of the number of medications, focuses on the appropriateness of the treatments. Inappropriate polypharmacy is defined as the prescription of medication under the following circumstances [18]: there is no clear evidence-based indication or if there was one in the past it is no longer applicable; doses are excessive; it fails to achieve the therapeutic objective; it has caused or may cause harm to an at-risk patient; or the patient cannot or does not want to continue with the treatment. This definition, while more useful on a case-by-case basis, makes it even more challenging to ascertain the true prevalence of polypharmacy.

Inappropriate polypharmacy in an elderly population with multiple pathologies is associated with adverse effects, being responsible for multiple visits to the ED. A proportional relationship exists between frailty and medication-related problems due to pharmacokinetic and pharmacodynamic alterations affecting drug absorption, distribution, metabolism, and excretion [19]. A 2018 systematic review involving 25 studies concluded that polypharmacy could contribute to the development of frailty in elderly patients [20]. Furthermore, it has been estimated that an individual taking five medications has a 58% probability of experiencing an adverse event, which increases to 82% with seven or more medications [21]. This poses a significant challenge for public health systems, leading to increased morbidity, mortality, ED visits, longer hospital stays, and future readmissions [22]. An English study revealed that 45% of patients seen in the ED were taking five or more medications. Among these patients, 39% were prescribed medications or combinations of medications that had the potential to cause adverse events. Most of these adverse events were linked to the hypotensive effects of antihypertensive drugs and serious interactions with anticoagulant therapy [23].

Another Dutch study, published in 2022, found that 43% of patients aged 70 or older who presented to the ED were taking between 4 and 9 medications, while 18% were taking 10 or more medications. Notably, polypharmacy, particularly severe polypharmacy (defined as more than 10 medications), was associated with a higher 30-day mortality rate. However, no significant relationship was demonstrated between polypharmacy and self-reported falls in the three months following discharge or with readmissions [24].

Given this escalating health concern, in 2017, the World Health Organization (WHO) launched its third global challenge for patient safety, “Medication Without Harm”. The objective of this initiative is to reduce the harm caused by medications by 50% within a five-year period, with a focus on three priority areas: high-risk situations, polypharmacy, and care transitions. Consequently, it is imperative to address polypharmacy through person-centered medication review programs that involve pharmacists as integral members of the healthcare team [25].

## 3. Impact of Drug-Related Problems on the Emergency Department

DRPs, defined as health problems that patients experience due to drug use or a lack thereof [26], constitute a major public health problem in Western countries. In the last two decades, several studies have been published pointing out the impact of DRPs on patients’ health and on the avoidable consumption of health resources. Lazarou et al. [27] ranked DRPs between the fourth and sixth cause of in-hospital death. DRPs have been associated with around 5–10% of hospital admissions [28] and 21% of hospital admissions, with close to 70% of cases considered to be preventable [29]. DRPs have also been associated with an increased risk of hospital readmission and early new ED visits, most of which are associated with widely used drugs such as cardiovascular, alimentary tract, and metabolism system medications [30].

The preventability of DRPs and their impact on ED visits have been studied specifically. Several studies and reviews have been published showing that that DRPs are consistently a frequent and avoidable cause of ED visits, and this has changed little over the decades. Patel et al. [3], by analyzing eight retrospective and four prospective studies, found that 28% of ED visits were related to DRPs. Of these, 70% were considered preventable, and as many as 24% resulted in hospital admission. Shehab et al. [4] found that the prevalence of ED visits due to DRPs in the United States was 4 per 1000 individuals in 2013 and 2014. The most common drug classes to be implicated were anticoagulants, antibiotics, diabetes agents, and opioid analgesics. Castro et al. [31] reported a prevalence of cases of DRPs, as primary or secondary diagnoses, leading to consultation of the ED of 37.4%, 71% of which were considered avoidable. These results regarding the prevalence and preventability of DRPs were not different from those reported in other healthcare settings [32], nor from those currently being published in our environment, where DRPs continue to be a prevalent and avoidable cause of consultation with EDs. In a recent multicenter study in Spain involving 4752 patients, it became evident that 366 (7.7%) visited the ED due to DRPs, with a prevalence ranging from 0 to 16.7% [33].

## 4. Strategies to Identify Patients with Drug-Related Problems

Given the growing complexity of the population, strategies to identify patients with DRPs play an essential role in healthcare systems, ensuring patient safety, optimizing medication therapy, and improving overall health outcomes [34].

Firstly, medication errors and adverse drug reactions can have serious consequences, including hospitalizations, morbidity, and even mortality. Detecting and addressing these problems early can minimize the potential harm to patients [35].

Secondly, these strategies are associated with an increase in the effectiveness of medication therapy. Recognizing issues such as drug interactions, therapeutic duplications, or suboptimal medication regimens can ensure that patients receive the most appropriate and effective treatment [36]. Avoiding medication errors and adverse events can reduce the economic burden on healthcare systems by preventing unnecessary hospitalizations, ED visits, and additional healthcare interventions [37,38].

Furthermore, the early identification of DRPs supports patient-centered care. It allows healthcare professionals to engage in shared decision making with patients, promoting patient empowerment and their involvement in their own healthcare. Detecting these problems enables healthcare providers to provide appropriate education, counseling, and support to patients regarding their medications.

Overall, implementing effective strategies to identify patients with DRPs is essential for enhancing patient safety, optimizing medication therapy, and improving healthcare outcomes.

Possible strategies to identify DRPs are as follows:

- Medication reconciliation: This strategy involves comparing the medications a patient is taking across different healthcare settings to identify any discrepancies, such as omissions, duplications, or dosage errors [39].
- Adverse drug event (ADE) monitoring: ADE monitoring systems aim to identify and report adverse reactions or events related to medications. This can involve electronic health records (EHRs), spontaneous reporting systems, or other surveillance methods [40].
- Prescription drug monitoring programs (PDMPs): PDMPs are electronic databases that track controlled substance prescriptions. They help to identify potential drug misuse, overuse, or “doctor shopping” behaviors [41].
- Medication review: Comprehensive medication reviews involve a thorough evaluation of a patient’s medication regimen, searching for potential DRPs such as interactions, inappropriate medication use, or adverse effects [42].
- Computerized decision support systems: These systems use algorithms and databases to provide healthcare professionals with real-time alerts, reminders, and recommendations regarding potential DRPs [43].
- Pharmacist-led interventions: Pharmacists play a crucial role in detecting and preventing DRPs. Their involvement in medication counseling, medication therapy management, and patient education can contribute to early detection and intervention [44].
- Clinical decision support tools: Computerized tools integrated into electronic health records (EHRs) that provide alerts and recommendations based on evidence-based guidelines, drug–drug interactions, or patient-specific factors [45].

## 5. Interventions to Reduce Readmissions Due to DRPs

ED revisits and hospital readmissions are common and represent a significant economic cost, being associated with a decrease in the quality of care. In fact, one of the proposed indicators for evaluating chronic care within national health system programs is hospital readmission, in addition to potentially preventable hospitalizations [46,47].

Programs to reduce hospital readmissions and new ED visits are widely recommended. One of the most popular programs is the “RARE campaign” (reducing avoidable readmissions effectively), focusing on five areas: comprehensive discharge planning, medication management (control/evaluation), engaging the patient and family, having a good connection with the next level of care, and improving communication during the transition [48]. Another project is “RED”, led by the Boston University Medical Center. This project focuses on redesigning hospital discharges to facilitate and streamline the process. Different techniques are used, such as discharge planning and scheduling follow-up appointments and tests, with a special focus on medication management and education regarding pharmacological treatment and medication reconciliation [49,50].

Hospital pharmacists can play a significant role in preventing hospital readmissions, as numerous publications have demonstrated. A healthcare organization that includes hospitals, primary care centers, and community pharmacies in Minnesota implemented a transition process to the outpatient setting, which included a medication review between clinical services by pharmacists. They conducted a study with data collected over two and a half years to examine the impact of comprehensive medication management (CMM) on readmission rates, demonstrating a decrease in readmissions within 30 days [51]. Kilcup et al. [52] conducted a similar study, calculating the savings achieved through the prevention of hospital readmission and thus supporting the hypothesis that medication assessment and reconciliation by clinical pharmacists within 3–7 days of hospital discharge reduce readmissions, leading to economic savings and improved patient safety.

Studies aiming to reduce hospital admissions by evaluating clinical pharmacist interventions are challenging to compare due to the variability in the types of interventions studied. This makes it difficult to determine which interventions are the most effective. However, several studies have demonstrated that the inclusion of pharmacists in a multidisciplinary team has a positive impact and improves clinical outcomes.

A randomized multicenter study conducted in Denmark [53] compared three different types of intervention. Patients were assigned to one of three groups: those receiving comprehensive pharmaceutical intervention (medication review; three motivational interviews; and follow-up by the primary care physician, pharmacist, and home nursing); those receiving standard care (no intervention); or those receiving basic pharmaceutical intervention (medication review). The recruitment period lasted for 20 months, and the included patients were followed for 6 months. The medication reviews and patient interviews were conducted in the hospital, and follow-up was carried out in collaboration with the patients’ primary care doctors. These results indicate a greater reduction in hospital readmission when interventions are more intensive, which is a strong indicator of a true intervention effect. Juanes et al. [54], in another randomized clinical trial, focused on those patients who visited the ED due to a DRP following a similar intervention (medication review, phone interviews after ED discharge, or follow-up by the primary care physician). They found a reduction in new hospital admissions 30 days after discharge [OR: 0.59, CI95%: 0.37–0.97].

## 6. Deprescribing Tools in Older Patients with Multimorbidity

Deprescribing is the process of withdrawing an inappropriate medication and is supervised by a health professional with the goal of managing polypharmacy and improving therapeutic outcomes [55]. The number of drugs that a patient is taking is the most important predictor of the adverse effects of polypharmacy [56]. Therefore, deprescribing has emerged as a nuclear intervention to optimize pharmacotherapy in older patients with multimorbidity.

Deprescribing interventions have been shown to significantly reduce potentially inappropriate medications (PIMs), potential prescription omission, and the incidence of adverse drug effects, in addition to improving medication adherence [57]. Despite the fact that there is a paucity of evidence when it comes to older people living with frailty and/or those with limited life expectancies, the available studies suggest that deprescribing could be safe, feasible, and well-tolerated, and may lead to important benefits in this population [58,59]. This must be accompanied by a high level of willingness to deprescribe among older people living with frailty [60]. Nevertheless, the lack of standardized definitions of PIMs for specific populations is a hindrance to the collection of evidence regarding deprescribing and a limitation for future implementation efforts [61].

Deprescribing tools that assist healthcare professionals in optimizing pharmacotherapy across a variety of chronic populations exposed to polypharmacy are increasingly available in daily practice. They are usually grouped as explicit criteria based on drug medication lists, or as person-centered frameworks which consider patients’ life expectancies, the time medicines take to show benefits, the goals of care, and patient values [62].

In terms of explicit criteria, more than 70 drug lists have been identified [63], including the Beers Criteria [64], the Fit-For-The-Aged (FORTA) [65], the Screening Tool of Older Person’s Prescriptions and Screening Tool to Alert to Right Treatment (STOPP/START) [66], and the STOPPFrail [67]. Explicit criteria might also be regrouped as either drug-oriented listing approaches (DOLAs) or patient-in-focus listing approaches (PILAs), for which a knowledge of some patient characteristics is required [63]. This taxonomy could be relevant because of the existence of differences in efficacy between DOLAs and PILAs when applied to deprescribing in older people [68].

Regarding person-centered frameworks, they represent an advanced type of medication review where patients’ values and preferences are taken into consideration, as well as their medication history and clinical data [68]. Either national or regional initiatives aiming to improve safety in patients with polypharmacy through person-centered framework have been developed [69]. This category includes the patient-centered prescription (PCP) model, a systematic four-stage process carried out by an interdisciplinary team, which centers therapeutic decisions on the patient’s global assessment. Such an approach, sensitive to the patient’s degree of frailty, has been associated with reducing inappropriate prescribing, medication burden, and 30-day hospital readmission, as well as improving medication adherence [54,62]. The PCP model was developed by the Central Catalonia Chronicity Research Group (C3RG), and its implementation for medication reviews in elderly and frail patients with multimorbidity is recommended by the Department of Health, Government of Catalonia (Spain) [70].

Although not classifiable in either of the two previous groups, scales for scoring anticholinergic and/or sedative exposure also aid in deprescribing due to the poorer clinical outcomes observed in older patients exposed to a high anticholinergic burden [71]. Several different scales have been developed, highlighting the Drug Burden Index (DBI) as the only scale that accounts for a patient’s dose. In addition, the DBI considers not only anticholinergic effects but also sedative effects. However, any scale is universally accepted [72].

Beyond the aforementioned tools, another approach to improving medication appropriateness and deprescribing is to use clinical decision support systems (CDSSs), which, combined with computerized physician order entry, have been shown to reduce drug prescription errors [73]. CDSSs link patient data with a knowledge base to generate information that helps clinicians to make decisions. Although the potential of CDSSs to reduce medication errors is clear, they are frequently underused [74]. Usually designed through relational databases, there is increasing interest in using ontology-based CDSSs in older patients to overcome major challenges such as a lack of interoperability or alert fatigue. Another challenge is related to designing automated deprescribing algorithms which are sufficiently faithful to the recommendations proposed by explicit criteria, or even by person-centered frameworks [75].

Furthermore, the frequency of medication reviews during which deprescribing is considered in older patients with multimorbidity is not uniformly recommended, but these reviews should ideally be performed on a regular basis, especially when it comes to patients with frailty or cognitively impaired older adults [68].

At this point, there are numerous tools providing guidance for deprescribing in older patients with multimorbidity, and these are valuable when assessing and improving medication appropriateness and adherence through medication reviews. Patient-centered approaches must be preferred due to their sensitivity to frailty status and patients’ values and preferences. In any case, it is necessary to gain more evidence regarding the benefits of these approaches in terms of patient-oriented outcomes, especially in those living with frailty and/or with limited life expectancies.

## 7. Limitations

The presented review has several limitations. Firstly, our examination of interventions involving various strategies to identify and prevent DRPs has yielded mixed or inconclusive data despite extensive study. While the aim of this review is to enhance understanding of DRPs in the ED and propose potential solutions to prevent new ED visits or hospital readmissions, the high heterogeneity among published studies and the diverse types of interventions conducted hinder the ability to establish a strong certainty regarding the most effective interventions. Secondly, the inherent nature of a narrative review poses a risk of subjective errors and interpretation. Thirdly, the review only encompasses Spanish and English studies, which introduces a potential bias in identifying high-quality studies related to drug-related problems in the ED.

## 8. Future Directions

Despite the well-established relationship between polypharmacy and healthcare resource utilization, the impact of DRPs on short-term outcomes in patients treated at EDs has not been thoroughly established. To date, there is a lack of solid evidence demonstrating how to identify patients with DRPs in the ED and whether targeting these patients improves long-term outcomes. Research into the use of machine learning models to screen ED patients for the likelihood of a DRP may help to alert ED physicians to identify those patients, improving patient´s medication review process. Predictive models may also prompt/help primary care providers to have more detailed discussions about the risks and benefits of starting new medications in high-risk patients.

Deprescribing medications in the ED setting presents challenges due to the brief nature of patient–clinician contact. However, conducting a comprehensive medication review seems reasonable for frail patients nearing the end of life, particularly those experiencing drug-related problems. Published guidelines advocating for the deprescription process in EDs emphasize considering patients’ opinions and establishing an effective communication system with physicians responsible for outpatient care as key elements in achieving effective treatment simplification and preventing DRPs. Future studies should investigate the effectiveness and applicability of the different tools presented when addressing patients with DRPs in the ED, including the evaluation of the intervention on patient´s readmissions, mortality, and quality of life.

## 9. Conclusions

DRPs are a common cause of ED visits. A progressive increase has become foreseeable in recent years due to the progressive aging of the population and the increase in polypharmacy.

**Figure 1:**
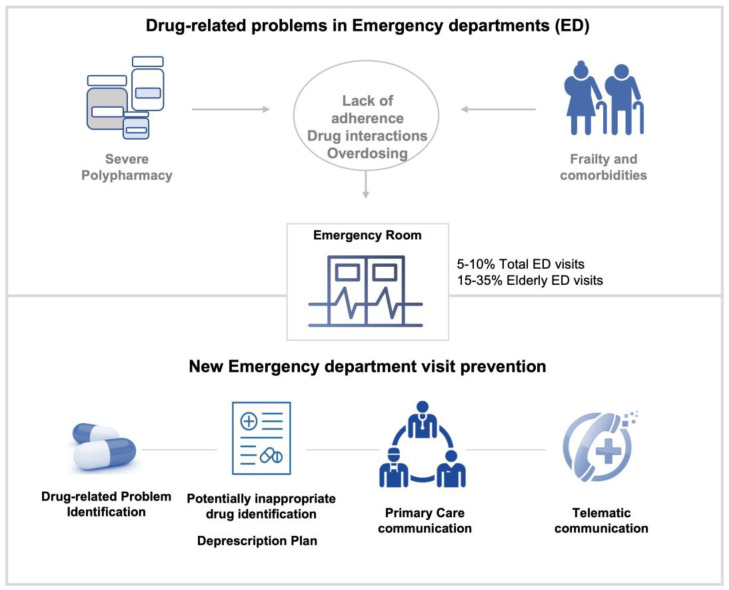
Main contributors and potential solutions for drug-related problems in emergency departments.

At this point, several studies focused on improving chronic treatment prior to ED discharge and ensuring fast communication with the patient’s primary care team have demonstrated a reduction in new hospital consultations. Thus, this is an essential intervention to improve patients’ quality of care, reduce ED visits, and minimize the saturation of Eds (Figure 1).

## Data Availability

The data presented in this study are available within the article or upon request from the corresponding author.
